# The Influence of Physical, Social, and Organizational Environments on Recreational Activities in Long-Term Care for Residents With Dementia: A Scoping Review

**DOI:** 10.1177/07334648251360098

**Published:** 2025-07-27

**Authors:** Ziying Zhang, Habib Chaudhury, Wenjin Wang

**Affiliations:** 1 Department of Gerontology, 1763 Simon Fraser University, Vancouver, BC, Canada; 2 Department of Landscape Architecture and Urban Planning, 14736 Texas A&M University, College Station, TX, USA

**Keywords:** assisted living, dementia, leisure, environment, staffing

## Abstract

Background: This scoping review explores how physical, social, and organizational factors influence recreational activity engagement for residents with dementia in long-term care settings.

Methods: A literature search in AgeLine, PsycINFO, CINAHL, Medline, and Web of Science identified studies on environmental factors affecting recreational activities for long-term care residents with dementia. A narrative approach was used to collate and summarize the findings from peer-reviewed English studies available until June 30, 2024.

Results: A total of 28 studies were reviewed, examining how physical, social, and organizational factors—such as homelike ambiance, staffing levels, and medicalized care culture—affect residents’ engagement in recreational activities. The review also highlights the interrelationship among these factors.

Discussion and Implications: The findings emphasize the importance of creating care environments that support activity participation. These insights can inform future assessments and the development of long-term care settings to improve activity experiences and outcomes for residents.

**What this paper adds**

• Summarizes evidence on how physical, social, and organizational environments shape recreational activity participation for residents with dementia in long-term care homes.
• Demonstrates that the physical, social, and organizational environments are interrelated in shaping residents’ ability to participate in activities.
• Emphasizes the benefits of a small household environment, where a homelike setting, familiar routines, and closer staff-resident relationships can encourage higher levels of participation in recreational activities.

**Application of findings**

• Informs design improvements in long-term care homes by implementing evidence-based modifications that create more accessible, homelike, and stimulating activity spaces, encouraging greater resident movement, independence, and participation in activities.
• Informs policies that embed recreation as a core component of dementia care and long-term care home, while fostering a supportive, flexible environment where staff can apply creativity and teamwork.
• Influences future research and policy by highlighting the need for more inclusive and person-centered approaches to activity programming in long-term care.

## Introduction

With the rapid growth of the aging population, an increasing number of people with dementia are being cared for in long-term care settings (World Health Organization, 2017). In this paper, long-term care (LTC) settings refer to residential care settings that provide 24-hour nursing or personal support for individuals with complex care needs, including nursing homes, assisted living facilities, and other residential care environments (Canadian Institute for Health Information [CIHI], 2022; Social Security Act, 42 U.S.C. §1395i–3, 2021). For simplicity, “LTC” is used to refer to these settings throughout the paper. Understanding the needs of residents with dementia and enhancing their quality of life has become a focal point for LTC providers and health authorities. For people with dementia, quality of life extends beyond meeting physical care needs; it is a multidimensional concept, including physical and psychological well-being, social interaction, and positive/negative affect (Klapwijk et al., 2016).

Recreational activities are key contributors to quality of life and well-being for residents with dementia living in LTC settings. These activities, defined as discretionary pursuits for entertainment, exercise, cognitive stimulation, creative expression, and socialization (Leitner & Leitner, 2012), take various forms including solitary activities (e.g., reading and watching television), physical activity, socializing, outings, hobbies, and programmed group activities (Gaugler & Kane, 2005; Hanson et al., 2014). In LTC settings, they can also be broadly categorized by who initiates them—either planned by staff or volunteers, or self-directed by residents based on their preferences and past roles. Participation in these activities benefits residents with dementia across several domains: utilizing remaining cognitive and physical abilities (Edvardsson et al., 2014; Palacios-Ceña et al., 2016), staying engaged in social interactions (Roland & Chappell, 2015), experiencing positive emotions (Smit et al., 2016), maintaining autonomy, and supporting personhood (Phinney et al., 2007). Despite these benefits, evidence suggest that residents’ needs for leisure are often unfulfilled, with most of their time spent unengaged or in activities that do not meet their interests (Palacios-Ceña et al., 2016; Smit et al., 2014; Wood et al., 2005). This inactivity can exacerbate neuropsychiatric and behavioral symptoms such as agitation, aggression, and apathy (Buettner & Fitzsimmons, 2003; Cohen-Mansfield, 2010).

Challenges related to activity engagement in LTC settings can be attributed to three main factors: residents’ personal characteristics, the content of activities, and the institutional environment (Buettner & Fitzsimmons, 2003; Smit et al., 2016; Tak et al., 2015). While the first two factors have been extensively studied, research on environmental influences remains relatively limited. Several literature reviews have begun to explore how environmental factors influence activity participation in residential care contexts. Plys (2019) reviewed recreational activities in assisted living settings, highlighting individual and environmental factors that influence participation. Anderiesen et al. (2014) conducted a systematic review on how physical environmental features support physical activity among residents with dementia in nursing homes. Narsakka et al. (2022) synthesized evidence on how the physical, social, and symbolic environments shape physical activity in LTC settings.

Building on previous reviews, this study aims to map the physical, social, and organizational environmental factors that have been examined in relation to recreational activities for residents with dementia in diverse LTC settings. This review is timely given the shifting priorities in long-term care toward holistic, person-centered models that recognize recreational engagement as central to quality dementia care.

## Methods

This scoping review aims to map current discourse and identify gaps in the literature. The study methods, which are described below, follow Arksey and O’Malley’s (2005) five-step scoping review framework, with methodological refinements recommended by Levac et al. (2010).

### Identifying the Research Questions

This review addresses two questions: (1) What physical, social, and organizational environmental factors have been examined in relation to residents’ engagement in planned and self-directed recreational activities in LTC settings? (2) What role do these factors play in influencing activity engagement?

### Identifying Relevant Studies

A comprehensive literature search was conducted to identify relevant studies aligned with the research questions. Five bibliographic databases were accessed in June 2024: MEDLINE (Ovid), the Cumulative Index to Nursing and Allied Health Literature (CINAHL) (EBSCO), PsycINFO (ProQuest), AgeLine (EBSCO), and Web of Science (Thompson Reuters). Keywords that were applied to the searching are listed in Table 1.

**Table 1.** Keywords for Searching in Databases.

Concepts	Keywords
Long-Term Care	Long-Term Care
	Nursing Home
	Residential Care
	Special Care Unit
	Assisted Living
Dementia	Dementia
	Alzheimer’s
	Memory Loss
	Cognitive Impairment
Activities	Leisure
	Recreational Activities
	Occupation
	Engagement
Environment	Environment
	Staff
	Policy
	Design
	Culture
	Practice
	Practitioner

### Selecting Studies

Covidence, an online systematic review management software (Veritas Health Innovation Ltd, Melbourne, Australia), was used to facilitate the screening process. Screening was limited to English-language, peer-reviewed journal articles published between January 1995 and June 2024. Two reviewers (ZZ and WW) independently screened the titles and abstracts of all retrieved citations using predefined inclusion and exclusion criteria (see Table 2). Articles identified as potentially relevant by either reviewer were advanced to the full-text screening stage. The full-text review was conducted by the first author, who assessed each article against the eligibility criteria. Uncertainties were resolved through discussion with the second reviewer (WW). The reference lists of included studies were also hand-searched to identify additional relevant sources. The selection process is summarized in the PRISMA flow diagram (Page et al., 2021, Figure 1).

**Table 2.** Inclusion and Exclusion Criteria.

Inclusion criteria	Exclusion criteria
• Focused on residents with dementia in long-term care settings providing full-time care	• Involved community-dwelling older adults, hospital patients, or residents living in facilities where full-time care was not provided
• Dementia status of residents may be identified through formal diagnosis (e.g., MMSE, clinical criteria) or inferred based on the care setting (e.g., dementia-specific care units)	• Focused solely on the impact of individual factors (e.g., gender) or the content of recreational activities (e.g., a specific activity program)
• Collected data from either the special care unit or regular care unit in the long-term care settings, and residents with dementia or their families or staff were identified as primary participants of the research	• Focused on physical activity, activities of daily living, self-care, or functional ability
• Investigated environmental aspects (physical, social, organizational) in relation to the recreational activity (programmed group recreational activities, one-one activities, household activities, and spontaneous recreational activities) of residents with dementia in long-term care settings	• Review studies, grey literature, book chapters, conference papers, dissertations, and news coverage

**Figure 1.**
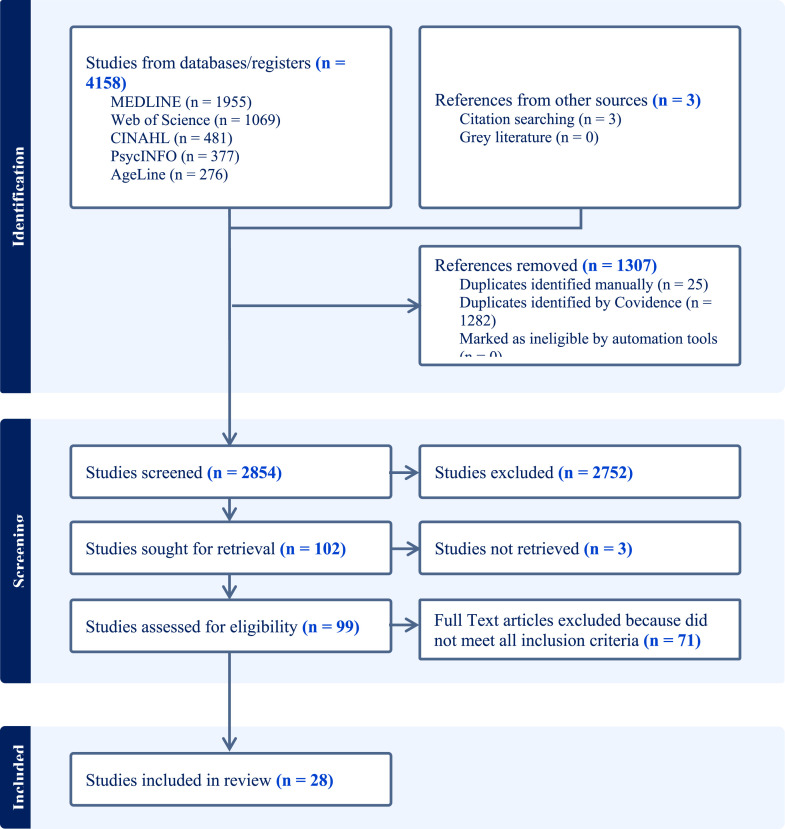
PRISMA flowchart.

### Charting the Data

A data-charting form was developed collaboratively by the two reviewers to guide data extraction. The form was piloted on a small number of included studies and refined iteratively to ensure consistency and clarity. The first author completed data charting for all included studies, extracting information such as the author(s), country of research, year of publication, study aim, methods, setting characteristics, and key findings about environmental features related to recreational activities. Ambiguities were discussed with the second reviewer (WW) to ensure consistency.

### Collating, Summarizing, and Reporting the Results

A structured narrative synthesis approach was used to organize and report the findings, in line with recommendations for interpretive synthesis in scoping reviews (Levac et al., 2010; Popay et al., 2006). Extracted data were grouped according to three predefined environmental domains: physical, social, and organizational. Within each domain, the various environmental aspects and key patterns were identified and summarized across studies. Cross-cutting insights and interrelations among environmental domains were also noted where relevant, to support a more integrated understanding of environmental influences.

## Results

### Study Selection

The electronic search yielded 4158 abstracts. After removing 1307 duplicates and 2752 irrelevant studies, 99 abstracts remained. Full-text screening led to the inclusion of 25 articles, with three additional articles identified through manual reference searches.

### Study Characteristics

A total of 28 articles were included in this review, spanning publications from 1999 to 2023. Studies were relatively evenly distributed across the USA, Europe, the UK, Australia, and Canada, with no representation from Asia, South America, or Africa. The studies covered a range of long-term care settings, including traditional nursing homes, dementia special care units, assisted living facilities, green care farms, and other household-model settings.

Nine studies employed qualitative methods, eleven used quantitative methods, and eight applied mixed-methods approaches. Qualitative studies commonly used interviews, focus groups, or ethnographic observation. Quantitative studies relied on structured tools such as Dementia Care Mapping (DCM; Brooker & Surr, 2005) or the Maastricht Daily Life Observation Tool (de Boer et al., 2017). Mixed-methods studies typically combined observational assessments with interviews or surveys, providing both behavioral and experiential insights.

### Findings

#### Physical Environment

The literature on physical environments primarily addressed general activity areas, with some studies also examining specialized spaces like kitchens, outdoor areas, and multisensory rooms. An emergent area of focus in the review was the household-model care setting, as a distinct physical environment relevant to resident engagement.

#### Activity Areas

Several studies examined how environmental stimulation affects resident engagement in activities. High background noise was identified as a distraction that reduces participation in group activities, while moderate noise levels were reported to be more engaging (Cohen-Mansfield & Jensen, 2022; MacDonald, 2006; Tak et al., 2015; Yous et al., 2023). Similarly, inadequate lighting, unpleasant odors, and excessive heat were described as barriers to participation (Cohen-Mansfield & Jensen, 2022; Tak et al., 2015). Background media and televisions tended to hinder rather than enhance interactions between residents and staff (Wood et al., 2005). In contrast, positive sensory elements—such as the sounds and smells of food from an open kitchen—were noted to encourage residents to initiate activities (Morgan-Brown et al., 2013).

Several studies highlighted that a homelike, object-enriched environment can enhance opportunities for residents to engage in activities (MacDonald, 2006; Smit et al., 2012; Voelkl et al., 2003; Wood et al., 2005). Recreational resources like musical instruments or a beach ball, along with personally meaningful objects such as photos, furniture, and art, and homelike amenities like a kitchen, were described as supporting engagement in both programmed and spontaneous activities (MacDonald, 2006; Richards et al., 2015; Rosteius et al., 2022; Voelkl et al., 2003; Wood et al., 2005). Conversely, the absence of recreational resources, such as books, puzzles, and storage, in living areas was associated with reduced engagement (Voelkl et al., 2003). Care environments that lacked a homelike ambiance often left residents feeling disconnected from the space and less likely to interact with objects (MacDonald, 2006; Voelkl et al., 2003).

The size and configuration of activity areas were another focus of the literature. Small, dedicated, and quiet spaces were reported to enhance engagement opportunities (Saperstein et al., 2004; Tak et al., 2015; Yous et al., 2023). Varied small activity spaces were considered crucial for both informal and formal activities, with smaller groups linked to stronger participation and social connection (Smit et al., 2017; Wood et al., 2005; Yous et al., 2023). Cohen-Mansfield et al. (2010) identified groups of four to nine people as optimal for engagement. However, some studies argued that activity areas must also be large enough to accommodate all residents, including wheelchair users, to ensure inclusivity (Cohen-Mansfield & Jensen, 2022; Richards et al., 2015).

Studies also examined other physical features of activity areas. Direct visual and physical access were found to be important for supporting participation (Schwarz et al., 2004; Voelkl et al., 2003). Long hallways were noted to challenge spatial navigation due to orientation difficulties and mobility limitations (MacDonald, 2006), and locked doors or dead ends were described as contributing to spatial disorientation (Richards et al., 2015). Appropriately designed hallways were seen as important not only for wayfinding but also for walking—a commonly preferred activity among residents with dementia (Tak et al., 2015). In addition, studies reported that the arrangement and design of furniture influenced opportunities for social interaction and spontaneous activities (Morgan-Brown et al., 2013; Voelkl et al., 2003).

#### Outdoor Space

Several studies described outdoor areas as important settings for both recreational activities and safe walking in LTC settings. The range of activities observed in these spaces included family visits, group activities (e.g., socialization, gardening, and barbecues), and spontaneous or individual activities (e.g., walking, watering the garden, and viewing plants) (Cohen-Mansfield & Werner, 1999; Cox et al., 2004; Giebel et al., 2022; Motealleh et al., 2022). These activities were noted to create a familiar link to past experiences for people with dementia, prompting memory recall (Motealleh et al., 2022).

Despite these benefits, outdoor spaces were frequently underutilized. Several studies attributed this to poor design, limited accessibility, and a lack of inviting or stimulating features (Cohen-Mansfield & Werner, 1999; Giebel et al., 2022). Weather is also a significant factor contributing to the underuse of outdoor spaces; staff have suggested adding shelters and other adaptations to accommodate different weather conditions and seasons (Cohen-Mansfield & Werner, 1999; Giebel et al., 2022).

To support outdoor engagement, several studies emphasized the importance of accessible and inviting environmental features. Physically and visually accessible designs were associated with more frequent outdoor activity. These included clear, short paths from living areas, wheelchair-accessible layouts, and unobstructed sightlines to outdoor areas from private or shared spaces (de Boer et al., 2017; Giebel et al., 2022; Motealleh et al., 2022; Rosteius et al., 2022; Schwarz et al., 2004). Design features identified as effective in encouraging activity and interaction included natural and sensory elements such as animals, diverse plantings, garden views, shaded seating areas, and overall aesthetic appeal; interactive amenities like raised flower beds; and spaces for socialization, including cafés and playgrounds designed to support intergenerational engagement (Cohen-Mansfield & Werner, 1999; Giebel et al., 2022; Motealleh et al., 2022; Rosteius et al., 2022).

#### Kitchen

Several studies identified the kitchen in LTC settings as a familiar environment for lifestyle-based activities and social interactions related to food preparation. In a questionnaire study of 631 LTC settings with unit kitchens, over half (53%) were part of the activity space, and 39% were located in dining areas (Marsden et al., 2001). Kitchen activities were primarily recreational, including baking, socializing, arts and crafts, holiday dinners, and ethnic meals (Marsden et al., 2001). Less commonly, household tasks such as meal preparation and housekeeping also took place in this setting (Marsden et al., 2001; Richards et al., 2015). These activities allowed residents to engage in routines they were familiar with, contribute to the care unit, and experience a sense of usefulness and homelike belonging (Marsden et al., 2001; Rosteius et al., 2022).

Kitchen design and accessibility were also discussed as key to fostering resident engagement and creating meaningful experiences. While visual access to the kitchen alone had limited impact, open physical access and opportunities for routine participation made the space more meaningful to residents (Saperstein et al., 2004). Effective kitchen design was described as balancing homelike aesthetics—such as wood cabinetry and greenery—with universal design features like wheelchair-accessible counters and open layouts to enhance usability and comfort (Marsden et al., 2001). Kitchen-based engagement consistently depended on staff facilitation, which became more challenging as residents’ functional abilities declined (Marsden et al., 2001; Rosteius et al., 2022).

#### Multisensory Environment

A multisensory environment (MSE) was described as being designed to stimulate the senses of sight, sound, touch, smell, and movement, using specialized equipment to offer activities tailored to the cognitive abilities of residents (Collier & Jakob, 2017; Cox et al., 2004). A survey of 16 LTC settings in England with MSE spaces reported that most rooms focused primarily on visual and tactile stimulation, using materials like bubble tubes and optic fibers, with fewer stimuli for proprioception, the vestibular system, or taste (Collier & Jakob, 2017). This was interpreted as indicating a need for more age-appropriate equipment that emphasizes reminiscence and familiar objects, which may enhance the effectiveness of these spaces. Some sensory elements, such as electrical devices, were noted to bring a sense of wariness or unfamiliarity (e.g., fiber optic sprays) to residents initially (Cox et al., 2004).

For residents, the MSE room was described as “something special,” providing a calming space for emotional release and privacy, and was considered by staff as a therapeutic alternative to medication (Cox et al., 2004). The room was reported to reduce passivity and encourage spontaneous engagement; staff observed that residents were hesitant to use the room at first but eventually “go their own way” and “take the lead” in activities (Cox et al., 2004). MSE rooms were viewed as particularly beneficial for residents in the later stages of dementia or those who were unable to participate in conventional activities (Collier & Jakob, 2017).

Despite the benefits, MSE rooms were often underused, with staff citing heavy workloads and lack of time as key barriers to their consistent implementation (Cox et al., 2004). Residents also frequently required staff guidance to enter the room and examples of how to handle the equipment (Cox et al., 2004). Additionally, staff highlighted the need for more training on how to support activities suited to varying resident abilities, in order to make the most of these spaces (Collier & Jakob, 2017).

#### Household Care Units

Several studies described household care units—typically accommodating 10 to 20 residents—as an emerging care model characterized by homelike design and person-centered practices. Key physical features include small household settings, private rooms, open-access kitchens, outdoor gardens, and open floor plans (de Boer et al., 2017; Richards et al., 2015; Rosteius et al., 2022; Schwarz et al., 2004). These elements encourage residents to come to common areas and engage in more diverse activities, particularly self-initiated and household ones (Adlbrecht et al., 2022; de Boer et al., 2017; Morgan-Brown & Chard, 2014; Richards et al., 2015; Rosteius et al., 2022; Smit et al., 2012, 2017).

#### Social Environment

The literature on social environments focused primarily on staff and family members, who play central roles in supporting resident engagement. A few studies also noted the broader community as part of the distal social environment.

#### Staff

Staff-resident interactions were consistently described as playing a critical role in facilitating or hindering recreational activities for residents with dementia. Consistent staff presence in common areas was reported to promote engagement in activities and interactions, as persons with dementia often rely on others to initiate participation (Adlbrecht et al., 2022; Morgan-Brown et al., 2013). Staff encouragement and social support for activity involvement were associated with higher rates of participation (Dobbs et al., 2005; MacDonald, 2006). Cox et al. (2004) referred to them as “the primary driver of positive affect” during activities. These interactions could include simple gestures, such as singing a song or reading the horoscope (Morgan-Brown et al., 2013). However, several studies found that staff often spent most of their time on care tasks involving limited resident contact (Morgan-Brown & Chard, 2014; Voelkl et al., 2003).

Barriers to activity provision were frequently linked to staffing-related constraints, including limited staff availability, high workload, and perceived job pressure (Ducak et al., 2018; MacDonald, 2006; Smit et al., 2017; Stoddart et al., 2024; Voelkl et al., 2003). These challenges were reported to contribute to fewer one-on-one interactions and small group activities (MacDonald, 2006; Saperstein et al., 2004), reduced implementation of innovative programs (Cox et al., 2004; Ducak et al., 2018), more passive engagement and reduced social interaction (Adlbrecht et al., 2022), as well as restricted use of space and fewer activity opportunities (Collier & Jakob, 2017; Giebel et al., 2022). Beyond availability, training and knowledge related to dementia and activity provision were identified as important for increasing staff competence and confidence in supporting engagement (MacDonald, 2006; Richards et al., 2015).

While most long-term care settings continue to rely on specialized staffing roles—such as assigning dedicated recreation or nursing staff—several studies have described the adoption of universal or multi-skilled staffing models, particularly in household-model environments (de Boer et al., 2017; Richards et al., 2015; Smit et al., 2012). Rather than dividing staff’s roles by function, this model combines tasks such as nursing care, food preparation, and recreational programming, sometimes extending to administrative duties like care planning (Smit et al., 2012). The universal model was found to enhance resident engagement in multiple ways. First, it was associated with increased staff presence and interaction in shared spaces, which created more informal and frequent opportunities for activity participation (Adlbrecht et al., 2022; Morgan-Brown & Chard, 2014; Morgan-Brown et al., 2013; Richards et al., 2015; Smit et al., 2012). Second, in smaller settings, staff developed greater familiarity with residents’ preferences and histories, enabling more personalized and responsive recreational support (Smit et al., 2012). Third, the model allowed for more flexible sequencing of tasks, supporting the integration of recreational moments into everyday routines rather than treating them as separate scheduled events (Richards et al., 2015; Rosteius et al., 2022). However, some studies raised concerns that the broad scope of responsibilities could overwhelm staff, potentially reducing their capacity to consistently initiate or coordinate recreational activities (Richards et al., 2015; Smit et al., 2012).

#### Families

Family involvement was also identified as a contributor to residents’ participation in recreational activities (Dobbs et al., 2005). First, families were reported to supplement staff by providing personalized, hands-on support for engagement, including social encouragement (Woodhall & Shaw, 2024). They could also perform supervisory tasks that were said to help residents participate more effectively (Rosteius et al., 2022). Second, families provided key information about residents’ personal histories, interests, and abilities, enabling activities to be tailored to individual needs (Dobbs et al., 2005; MacDonald, 2006; Woodhall & Shaw, 2024; Yous et al., 2023). Third, families played a role in vigilance and advocacy by identifying and addressing deficiencies in activity provision (Woodhall & Shaw, 2024).

Several studies highlighted gaps in coordination between families and staff in relation to residents’ care, particularly in aligning their approaches and expectations. In a focus group study, both staff and family members emphasized the need for greater mutual understanding and education about dementia (MacDonald, 2006). Activity staff also reported encountering resistance from families and internal groups when implementing small group activities, as stakeholders expressed a preference for programs that “get them [residents] all involved” (Ducak et al., 2018). Additionally, while staff recognized the potential value of increased family involvement in the multisensory room, they noted that it remained underutilized—largely due to limited staff encouragement and families’ lack of familiarity with the space (Collier & Jakob, 2017).

#### Broader Community

Being in contact with the broader community—such as through outings to public facilities, interactions with community members, and visits from external groups—was described as enriching the activity experiences of residents (Tak et al., 2015). However, such engagement was reported to require support from LTC settings, including transportation, which was not always readily available (Tak et al., 2015). In addition, volunteers were noted to provide valuable assistance by supporting both general and specialized activity programs (Ducak et al., 2018; Smit et al., 2017; Stoddart et al., 2024).

#### Organizational Environment

The literature on organizational environments focused primarily on care and organizational culture. Fewer studies noted the impact of activity programming and resource allocation in LTC settings.

Several studies described the prevailing culture of care in LTC settings as emphasizing clinical efficiency over residents’ quality of life. This often-reinforced job divisions and staff hierarchies, in which nursing roles were prioritized while recreational roles were comparatively undervalued (Ducak et al., 2018; Richards et al., 2015; Stoddart et al., 2024). These disparities were reported to lead to disagreements between departments over care philosophy, which in turn hindered nursing staff from engaging residents in recreational activities (Voelkl et al., 2003). In one study, researchers observed residents being interrupted during music sessions for personal care tasks (Richards et al., 2015). Additionally, activity staff were noted to receive little support from other departments and had limited input into residents’ care plans (Ducak et al., 2018). Cox et al. (2004) suggested that a more collaborative care culture—one that bridges disciplinary silos—could better support engagement and resident well-being.

The highly regulated nature of LTC was another barrier to meaningful engagement. Staff were often focused on meeting external compliance targets tied to funding, leaving limited time and flexibility for facilitating activities (Ducak et al., 2018; MacDonald, 2006). For example, while chores were recognized as potentially meaningful, staff often completed them independently due to concerns about breaching regulations. Transformative administrators were described as instrumental in promoting a culture that valued activity engagement by prioritizing time for recreation, fostering welcoming environments, and embedding activities into daily routines (Rosteius et al., 2022; Saperstein et al., 2004; Smit et al., 2017; Stoddart et al., 2024).

In many LTC settings, activities were commonly organized through centralized programs and fixed schedules (Richards et al., 2015; Smit et al., 2014, 2017; Tak et al., 2015). This approach was widely reported to limit resident choice and participation (Smit et al., 2014, 2017; Tak et al., 2015), though some residents were found to appreciate the structure, as it helped them know what to expect and encouraged them to come to common areas (Tak et al., 2015; Voelkl et al., 2003). In contrast, some household-model environments were observed to be moving away from fixed schedules, encouraging spontaneous, interest-driven, or one-on-one activities that were embedded into the flow of daily life (Richards et al., 2015; Rosteius et al., 2022; Smit et al., 2014). In these settings, residents and staff were empowered to co-decide on activity plans, promoting engagement and a sense of shared living (Richards et al., 2015; Rosteius et al., 2022; Smit et al., 2012).

The success of activity provision was also linked to the availability and flexibility of key resources, such as transportation, physical space, and materials (Ducak et al., 2018; Tak et al., 2015). Funding levels were noted to affect program offerings, staffing ratios, and opportunities for training staff in activity facilitation (Ducak et al., 2018).

### Interrelationship Among the Three Factors

A recurring observation across studies was that well-designed physical environments did not always support engagement on their own, particularly when social or organizational supports were lacking. Staff were consistently described as playing a crucial role in realizing the value of these spaces, shaping the extent to which physical environments could enable meaningful activity participation (de Boer et al., 2017; Rosteius et al., 2022). Low staffing levels were reported to hinder the effective use of various activity spaces (Cohen-Mansfield & Werner, 1999; Cox et al., 2004; Giebel et al., 2022; Saperstein et al., 2004), and limit meaningful interaction with environmental objects (Wood et al., 2005). Heavy workloads and limited time also constrained staff capacity to facilitate activities in multisensory rooms (Cox et al., 2004). Moreover, lack of staff familiarity with space features was a barrier to effective use (Collier & Jakob, 2017; Rosteius et al., 2022). Environmental features alone do not ensure engagement, several studies emphasized the importance of intentional planning and integration with programming to fully leverage the potential of a well-designed space (Cox et al., 2004; Schwarz et al., 2004; Wood et al., 2005).

Beyond individual staff practices, the organizational environment shapes how physical spaces are used—not through design changes, but via institutional policies and daily routines. For example, a national survey in the United States found that 69% of facilities with outdoor areas required staff accompaniment, which increased operational costs and limited resident access (Cohen-Mansfield & Werner, 1999). Similarly, LTC settings may determine whether kitchens remain open to residents and whether staff are permitted to leave tasks (e.g., washing a cup) unfinished to encourage resident participation (Rosteius et al., 2022). To better align environmental design with care philosophies and institutional goals, Saperstein et al. (2004) recommended collaboration between designers and care providers during the planning process.

## Discussion

This review aimed to explore and collate evidence to create a comprehensive understanding of the physical, social, and organizational dimensions of the environment related to the recreational activities of residents with dementia in LTC settings. A table (Table 3) has been added to outline key environmental factors, with dementia-specific features emphasized in bold for clarity.

**Table 3.** Key Environmental Features.

Environmental factor	Key features	Impact on recreational engagement
Physical	**Small household units**	Encourages a sense of familiarity and autonomy
	**Stimulation management**	Reduces sensory overload and enhances comfort
	**Presence of recreational resources**	Supports self-directed engagement in meaningful and familiar pastimes
	Open-access kitchens	Facilitates reminiscence, sensory engagement, and social interactions
	Direct visual and physical access to activity spaces	Encourages passive and active participation by making spaces more inviting
	Outdoor spaces	Provides an accessible and stimulating environment for nature-based activities
	Multisensory environments	Improves engagement through sensory stimulation
	Furniture layout	Fosters social bonds and group cohesion, reducing isolation
	**Spatial orientation**	Improves independent navigation and reduces confusion
Social	**Staff consistent presence**	Enables continuous engagement, reducing passive behaviors
	**High staff-to-resident ratio**	Allows for greater one-on-one and small-group interaction
	**Dementia-specific communication techniques**	Ensures staff can effectively support engagement based on dementia-specific needs
	**Staff encouragement and facilitation**	Encourages spontaneous activity participation without needing structured programming
	Families’ involvement	Supports personalization of activities, increasing meaningful engagement
	Volunteers and community	Broadens social opportunities and introduces fresh activity dynamics
	Staff role flexibility	Creates a more dynamic care model where staff facilitate informal engagement
Organizational	**Flexible schedule**	Allows residents to choose participation based on preference
	Administrative policies	Removes unnecessary administrative barriers to meaningful engagement
	**Interdisciplinary collaboration**	Enhances teamwork in facilitating resident activities
	Funding	Ensures sustainable support for a variety of recreational opportunities
	Shared decision-making	Encourages resident agency in choosing leisure activities
	Reduce task-oriented care	Shifts focus from task-oriented care to holistic well-being

Considering the physical environment, accessibility was the most consistently reported feature influencing residents’ active living and use of various activity spaces. Reviewed studies highlighted accessibility in three domains: physical (clear orientation and unobstructed pathways for all residents) (Motealleh et al., 2022), visual (direct sightlines to activity spaces from residents’ rooms and common areas) (Schwarz et al., 2004; Voelkl et al., 2003), and temporal (free access to activity spaces throughout the day) (Rosteius et al., 2022; Saperstein et al., 2004). Residents coming to and staying in common areas encourages further participation in activities (Adlbrecht et al., 2022; Morgan-Brown et al., 2013). Collectively, these forms of accessibility support not only participation but also residents’ sense of autonomy—by allowing them to navigate spaces independently, access preferred areas at will, and initiate activities on their own terms.

This review identified three specific activity areas: outdoor spaces, kitchens, and multisensory environments (MSEs). To our knowledge, their effects on recreational activities have not been examined or discussed in previous reviews. These environments were reported to hold potential for encouraging activity engagement, as they can draw residents into activities that were familiar to them in their earlier lives (Cox et al., 2004; Motealleh et al., 2022). However, the use of these environments often depends on staff facilitation and environmental features. Kitchen and MSE activities require staff to assess residents’ abilities, guide participation, and tailor the experience (Cox et al., 2004; Marsden et al., 2001; Rosteius et al., 2022). Familiar, inviting, and sensorially stimulating features, such as homelike kitchens or plant-filled gardens, can foster engagement (Marsden et al., 2001).

Staffing levels were consistently identified as a key determinant of the adequacy of social interaction, activity provision, and the utilization of activity spaces (Ducak et al., 2018; MacDonald, 2006; Smit et al., 2017; Stoddart et al., 2024; Voelkl et al., 2003). Training in dementia care and recreational programming helps staff communicate effectively, tailor activities to varying capacities, and create opportunities for engagement (Collier & Jakob, 2017; Cox et al., 2004; Marsden et al., 2001; Rosteius et al., 2022). These findings highlight the need for ongoing, context-specific training to support individualized engagement. Families are also important social contributors to residents’ well-being and activity engagement. Close collaboration between families and staff can enhance personalized care, foster mutual understanding, and expand the range of meaningful engagement opportunities.

Organizational factors, particularly care culture, shape the values, practices, and working conditions that influence recreational engagement. The literature described how the medicalized orientation of many LTC settings, along with philosophical divides among staff, can constrain recreation practitioners’ ability to provide person-centered activities (Ducak et al., 2018; Stoddart et al., 2024; Voelkl et al., 2003). These tensions often reflect entrenched hierarchies that prioritize nursing duties over social care. Leadership plays a crucial role in shifting these dynamics by supporting interdisciplinary collaboration and a more flexible work environment. Such changes enable staff to spend more time with residents, build meaningful relationships, and integrate engagement into daily routines.

The findings of this review support existing literature on the interdependence of physical, social, and organizational environments in shaping engagement outcomes (Benjamin et al., 2014; Narsakka et al., 2022). This interaction is particularly evident in the use of kitchens and outdoor areas, where well-designed spaces often remain underused due to staffing constraints, institutional routines, or risk-averse policies (Cohen-Mansfield & Werner, 1999; Collier & Jakob, 2017; Saperstein et al., 2004). While previous reviews have noted these interconnections, the present study offers a more detailed account of the mechanisms through which these interactions operate in everyday practice. Notably, this review found a heavier concentration of literature on physical environments compared to social and organizational domains. However, as many studies have noted, the effectiveness of physical features depends on social facilitation and institutional support. This disconnect points to a need for future research to move beyond isolated environmental domains and examine how structural and relational factors enable or constrain the use of space in practice.

Reflecting the need for integrated approaches, a growing body of literature has focused on household-model care environments, which integrate physical, social, and organizational elements—often through small-scale units, personalized design, universal staffing, and shared decision-making—to support resident engagement. Studies suggest that these environments support greater activity participation by embedding smaller-scale, interest-driven routines into daily life and tailoring them to residents’ personal histories, preferences, and capacities (de Boer et al., 2017; Morgan-Brown & Chard, 2014; Schwarz et al., 2004). We therefore recommend adapting these strategies in LTC settings to enhance activity engagement and improve overall resident well-being.

Many reviewed studies focused on staff-led or structured activities, with limited attention to resident-initiated or spontaneous forms of engagement—for example, playing puzzles or gardening without prompting. Future research should explore how spatial, material, and organizational features can be optimized to support these forms of engagement that reflect residents’ personal interests and choices. In parallel, this review identified three types of physical features—built, ambient, and interior. While most studies emphasized built elements such as layout and accessibility, fewer examined how ambient conditions (e.g., light and temperature) or interior elements (e.g., familiar objects) shape sensory experiences and influence residents’ activity participation. These latter aspects may be especially amenable to low-cost or small-scale modifications, offering practical opportunities to enhance meaningful engagement.

### Implications

This review synthesized evidence on three key environmental domains that influence recreational activity engagement, offering actionable strategies for LTC administrators and policymakers to improve care environments. A checklist (Table 4) outlining key areas and actionable strategies has been generated for LTC administrators to refer to when reflecting on their existing resources and amenities. These strategies are interpreted as implications based on the findings from this review. A few issues raised by the studies pertain to broader systemic and societal topics, such as macro-level policies, regulations, ageism, practitioner education, and labor shortages. Policymakers should reassess the role of recreational activities in LTC settings, focusing on environments that promote active living. Key policy changes include enhancing activity-friendly spaces, improving recreation therapy training, and adopting more person-centered regulations.

**Table 4.** Actionable Strategies for LTC Administrators and Staff.

Key areas	Actionable strategies
Physical	
Accessibility	Ensure clear, easy-to-navigate pathways and direct sightlines from rooms to activity spaces. Allow free access to these areas throughout the day
Stimulation management	Control temperature, noise, lighting, and odors to create a comfortable, engaging atmosphere for activities
Recreational resources	Provide a variety of easy-to-access recreational items that cater to different interests
Homelike ambience	Add familiar household items and personal objects to create a welcoming, homelike environment
Universal design	Use designs that promote level of accessibility and interactivity for all residents, including those with physical or cognitive challenges
Social	
Staff	Ensure enough staff to allow time for meaningful interactions. Train staff on dementia care and activities
Family	Involve family in planning personalized activities and improve communication between staff and families
Community	Encourage community volunteer programs to support activities and create intergenerational engagement
Organizational	
Culture	Reduce divisions between departments, promote communication, and empower activity staff into care planning
Activity programming	Offer a mix of group and individual activities to accommodate residents’ preferences
Policy	Update policies to promote active living environments and encourage staff to integrate activities into daily routines

Although there is considerable literature on the impact of LTC environments on recreational activities, significant gaps and limitations remain that warrant further investigation. Regarding the physical environment, our understanding of how the design of activity spaces influences residents’ engagement is still limited. To inform decisions on design new or retrofit existing physical environments, future research should move beyond general recommendations and identify specific environmental parameters—such as level of lighting—that can effectively support engagement.

For social and organizational aspects, there is a notable gap among the included studies between the specialized and universal staffing mode in providing recreational activities. Future studies can explore how staff roles and responsibilities revolve around support, encouragement, and initiation of activities, as well as how coordination between staff groups can be better achieved. Additionally, existing research do not address administrators’ perspectives on recreational activities and how organizations can support practitioners’ work. As many barriers mentioned in the extant literature regarding activity provision are related to care philosophy and organizational structures, administrators’ understanding and perspectives are key to reforming LTC policies and practices.

Considering the methodology, the reviewed literature employed a relatively balanced mix of qualitative, quantitative, and mixed-methods approaches. Quantitative studies typically used observational tools to assess residents’ engagement, focusing on aspects such as level, duration, and attitudes. Qualitative research explored various perspectives on recreational activities and care environments. Mixed-methods studies were widely used to evaluate specific environments, such as gardens or non-traditional care units. However, in the 17 qualitative or mixed-method studies that included interviews or focus groups, participants were predominantly staff and family members, leaving the perspectives of residents largely underrepresented. Residents’ insights are crucial for informing person-centered design. Future research should also engage more culturally and ethnically diverse populations to better reflect the heterogeneous nature of LTC settings (Chaudhury et al., 2013).

### Limitations of the Review

There are a few limitations in this review. First, only peer-reviewed research-based articles were included in the study. Since LTC personnel and practice are highly policy-relevant topics, grey literature (e.g., regulations) can better contribute to understanding the policy and processes related to recreational activities in LTC settings. Second, research on the use of technology was not included in this review, as it represents a distinct and expanding field that warrants dedicated exploration. Its classification between environmental factors and interventions remains ambiguous, further justifying its exclusion from this review.

## Conclusion

This review provides comprehensive evidence on the impact of the environment from physical, social, and organizational domains, emphasizing the need for an integrated, holistic approach to support meaningful and sustainable activity engagement. It identifies underexplored areas such as resident-initiated activity and household-model environments. Future research and practice should focus on designing and adapting LTC environments that enhance residents’ opportunities for, and experiences of, meaningful activity engagement.

## Supplemental Material

Supplemental Material - The Influence of Physical, Social, and Organizational Environments on Recreational Activities in Long-Term Care for Residents With Dementia: A Scoping Review Supplemental Material for The Influence of Physical, Social, and Organizational Environments on Recreational Activities in Long-Term Care for Residents With Dementia: A Scoping Review by Ziying Zhang, Habib Chaudhury, and Wenjin “Summer” Wang in Journal of Applied Gerontology
