# Supplementary material for: The Influence of Physical, Social, and Organizational Environments on Recreational Activities in Long-Term Care for Residents With Dementia: A Scoping Review
Source: J Appl Gerontol. 2025 Jul 27;45(6):1136–48. doi: 10.1177/07334648251360098 (PMC13133420; doi:10.1177/07334648251360098)
Supplement: Supplemental Material - The Influence of Physical, Social, and Organizational Environments on Recreational Activities in Long-Term Care for Residents With Dementia: A Scoping Review [file sj-pdf-1-jag-10.1177_07334648251360098.pdf]

Supplemental material for The Influence of Physical, Social, and Organizational Environments on Recreational Activities in Long-Term Care for Residents with Dementia: A Scoping Review

Table S1. Data Chart of Articles Included in the Scoping Review

First Author<br>Year<br>Country	Objectives	Methods	Setting	Main findings for the relationship of environment and activity
Adlbrecht<br>2021<br>Austria	Assessed the impact of the Specialized Care Unit care model on activity engagement and social interaction among individuals with dementia, comparing these outcomes to those in traditional nursing homes.	Quantitative<br>The Maastricht Daily Life Observation Tool	Three long-term care homes: one dementia special care units<br>two traditional nursing homes	Residents of special care units had a significantly higher chance for engagement in activities and social interaction.<br>Environmental factors that promote the engagement in activities and social interaction in SCUs are:<br>• High staff presence<br>• The longer time periods residents of SCUs spent in communal areas, where engagement is more likely to take place.
Cohen-Mansfield<br>1999<br>USA	Describe the experience of long-term facilities with outdoor areas.	Quantitative<br>Questionnaires with LTC staff	320 long-term care facilities with special care units	Factors influencing the use of outdoor spaces:<br>• Weather-related problems<br>• Accessibility<br>• Design<br>• Supervision
Cohen-Mansfield<br>2010<br>USA	Examine the impact of setting characteristics and presentation effects on engagement.	Quantitative<br>Observational Measurement of Engagement	Seven nursing homes	Participants were engaged more often with moderate levels of sound and in the presence of a small group of people (from four to nine people).
Cohen-Mansfield<br>2022<br>USA	Clarify the presence and types of barriers to group activities for persons with dementia	Mixed- Method<br>Group Observation Measurement of Engagement<br>Interviews with therapeutic recreation staff and research observers	Six nursing home units	Environmental barriers:<br>• noise from the outside,<br>• lack of space<br>• schedule conflict<br>• uncomfortable environment
Collier<br>2017<br>UK	Identify what equipment care homes were using in their sensory spaces and staff response to using these room designs.	Qualitative<br>Semi-structured interviews & Observations	16 care homes	Factors influencing the use of Multisensory Environment:<br>• Not knowing what to do in the room,<br>• Design and setting up of the space<br>• Including relatives and care staff.
Cox<br>2004<br>Australia	Examine the effectiveness of two types of multisensory environments, a Snoezelen room	Mixed- Method<br>Affect Rating Scale (ARS).	One care home	Residents experienced increased **positive affect**, such as pleasure and contentment, in all three environments (Snoezelen room, garden, and living room)

	and a landscaped garden, in improving the wellbeing of older individuals with dementia, and compare the effect of these environments with their usual living room environment.	Interviews with six caregivers and six visitors.		**Snoezelen room** was associated with relaxation and calming effects, while the **garden** promoted animation and engagement in residents.<br>**Caregivers and visitors** reported that both environments had a positive impact on their own well-being, enhancing their work experience and providing therapeutic benefits beyond the traditional care setting.<br>The **one-on-one care** provided by caregivers was the primary driver of positive affect, regardless of the specific environment.
de Boer<br>2016<br>Netherlands	Examine whether residents of green care farms are more engaged in (physical) activities and social interaction than are residents of traditional nursing homes and regular small-scale living facilities.	Quantitative<br>The Maastricht Electronic Daily Life Observation tool	Five green care farms<br>Four traditional nursing homes<br>Nine regular small-scale living facilities	Residents of green care farms were:<br>• Significantly more physically active than residents of regular small-scale living facilities,<br>• Significantly less often engaged in passive/purposeless activities compared with residents of traditional nursing homes,<br>• Had significantly more active engagement<br>• Came outside significantly more than residents of traditional nursing homes.
Dobbs<br>2005<br>USA	Describes the characteristics associated with activity involvement	Quantitative<br>Patient Activity Scale–Alzheimer's Disease	45 assisted living facilities and nursing homes	Activity involvement was related to family involvement in care and staff encouragement, after adjusting for resident age, gender, race, cognitive and functional status, and comorbidity.
Ducak<br>2016<br>Canada	Investigated recreation staff and multidisciplinary consultants' perceptions of factors that affected implementing Montessori Methods for Dementia in long-term care homes	Qualitative<br>Semi-structured interviews with 17 participants who worked in these homes.	NA	Limiting Factors<br>• Regulating and Funding Medical Practices<br>Enabling Factors<br>• Educating and Understanding<br>• Seeing Results is Believing<br>• Being Supported<br>LTC Culture Change Tensions<br>• Shifting Practice Amidst Resistance to Change
Giebel<br>2022<br>UK	Involve family members and care home staff in developing a meaningful garden space in a care home	Qualitative<br>Focus group with staff members<br>Interview with family carers	One care home	Factors that useful in the garden for the residents to meaningfully engage:<br>• Current opinion on physical environment<br>• Access<br>• Adaptation to the environment<br>• Staffing<br>• Socialising<br>• Sensory features<br>• Active meaningful participation
MacDonald<br>2006	Investigate how the institutional environment impacts leisure	Qualitative<br>Focus groups with family	One care home	Family members reported to restrict opportunity for leisure:<br>• Lack of staff, routine care

Canada	opportunities and choices of individuals with Alzheimer's disease in a long-term care facility, from the perspectives of staff members and family caregivers.	members and staff		• Lack of family involvement<br>• Physical environment constraints<br>Staff reported to restrict opportunity for leisure:<br>• Lack of staff, routine care<br>• Insufficient knowledge of residents<br>• Staff-related issues, environmental issues<br>• Discrimination of individuals with Alzheimer's disease<br>• Lack of family support and education
Marsden 2001 USA	Identify physical features that are typically included in therapeutic kitchen design and to explore how these features support daily use for residents and staff in relation to food service systems and activities programming.	Mixed- Method<br>Observation and semi-structured interviews with staff<br>Questionnaire from 85 facilities	Four care homes<br>85 facilities	Universal design features should be incorporated to a greater extent and certain features are more common, reinforce homelike imagery, or enhance safety.<br>A higher number of residents participate in more recreational activities, such as baking, than they do in household chores, such as meal set-up, and therapeutic kitchens are not always linked to food service systems.
Morgan-Brown 2012 Ireland	Study the effect of the change to a household model unit on residents' interactive occupation and social engagement and develop a research protocol which could quantitatively measure a whole room environment.	Quantitative<br>Assessment Tool for Occupation and Social Engagement	Two nursing homes	Residents spent more time in the communal living spaces and were more likely to be active and engaged in the household model units. They spent this time being more socially engaged, being more interactive with their environment and doing more for themselves.
Morgan-Brown & Chard 2014 Ireland	Compares the Social Engagement and Interactive Occupation of residents with dementia in two Irish nursing homes, before and after conversion to a household model environment.	Quantitative<br>Assessment Tool for Occupation and Social Engagement	Two nursing homes	Changing from a traditional to a household model nursing home increased the interactive occupation and social engagement of residents, staff, and visitors within the communal living areas.<br>The role of a homemaker in the household unit was critical for maintaining residents' participation and engagement, including engaging in familiar everyday domestic tasks.
Motealleh 2022 Australia	Investigate the effect of a garden improved according to dementia-friendly environment characteristics on agitation, apathy, and engagement of people with dementia in one residential aged care facility	Mixed- Method<br>Outcome measurement tools<br>Semi- structured interviews<br>On-site observations	One care home	Characteristics of dementia friendly garden that influence activity engagement:<br>• The presence of sensory-provoking elements in the garden<br>• Meaningful engagement in the garden<br>• Accessibility of the garden
Richards 2015 Australia	To observe the opportunities for occupational engagement of persons living with dementia in a traditional vs. a non-traditional	Mixed- Method<br>The Residential Environment Impact Survey, including a visual	One non-traditional facility<br>One traditional residential facility	The non-traditional facility provided additional opportunities through employee interactions and features of the physical environment.<br>Interviews revealed six themes:<br>• Comfortable environment

	dementia facility in rural Australia.<br>Investigate the perceptions of OE by the employees of each type of facility in rural Australia.	walk-through of the facility; Observation of three activities; Interviews with the residents; Interviews with organisational representatives		• Roles and responsibilities<br>• Getting to know the resident<br>• More stimulation can elicit increased engagement<br>• The home-like experience<br>• Environmental layout.
Rosteius<br>2022<br>Netherland	Analyze the care environment of green care farm based on their physical, social and organizational context.	Mixed- Method<br>Ethnographic observations<br>Interviews with residents, family members, and staff members<br>Focus group with staff members<br>OAZIS-dementia tool	A green care farm care home	Four themes were identified as crucial during daily life on the green care farm<br>• Stimulating the senses<br>• Engaging in purposeful activities<br>• Sharing responsibilities<br>• Creating a community in a new home<br>The physical environment encouraged and facilitated meaningful in-/outdoor activities and social encounters.<br>The organizational environment supported the use of the physical environment by aligning processes and transporting the vision.<br>The social environment focused on collaboration and creating a home-like atmosphere by including residents in household- and farm chores.
Saperstein<br>2004	Summarize 4 of the most predominant disconnects observed and explore the perspectives of facility staff and administrators in these areas.	Qualitative<br>Observation	Six long-term care facilities	Residential kitchens—Enhancing homelike aesthetic and maintaining resident skills<br>Residential size spaces—Enhancing resident engagement in socialization and activities
Schwarz<br>2004<br>USA	Assess the influence of design interventions on an existing long-term care facility for residents with dementia and the staff members.	Mixed- Method<br>Environmental assessment<br>Behavior mapping,<br>Focus groups with staff members	One Nursing home	After the renovation of the facilities, they were assessed to be of better quality, and residents participated more in programmed activities. Due to lack of appropriate activities, high staff turnover, and family members' resistance to the relocation of their loved ones the design did not meet all the behavioral expectations.
Smit<br>2012<br>Netherland	(1) In what way is small-scale care related to overall activity involvement of residents with dementia? (2) To what extent is small-scale care related to the involvement of residents with dementia in different types of activities?	Quantitative<br>Dementia Care Mapping	136 long-term care facilities	Residents of care facilities with more group living home care characteristics were more involved in overall and preferred activities. Furthermore, they were involved in more diverse activities. Overall, no relationship was found between the number of residents at the facility and activity involvement.
Smit<br>2014	Explore residents' involvement in different types of occupation and	Quantitative<br>Dementia Care Mapping	136 long-term care facilities	Long-term care facilities that did so more frequently generally had a more homelike atmosphere, supported social interaction through the

Netherland	its relation to wellbeing, and those characteristics of care facilities that might facilitate occupation among residents.			environment, and had no central activity program.
Smit<br>2017<br>Netherland	Clarify factors predict higher activity involvement.	Quantitative<br>Dementia Care Mapping	136 long-term care facilities	The most important predictors of higher involvement were: absence of agitation, less ADL dependency, and a higher cognitive status of the residents, higher staff educational level, lower experienced job demands by care staff and a smaller number of residents living in the dementia care wards of a facility. More social supervisor support as perceived by staff was found to predict less activity involvement.
Stoddart<br>2022<br>Australia	Explore personal care attendants' experiences and understandings of their role in leisure provision to identify the barriers and enablers that may exist when working alongside people living with dementia in residential aged care facilities.	Qualitative<br>Focus groups with personal care attendants	Three residential aged care facilities	Barriers to leisure provision<br>• Workloads and prioritisation of physical care over leisure<br><br>Enablers to leisure provision<br>• Experience-based knowledge of staff<br>• Perceptions of leisure<br>• Organizational support and resourcing
Tak<br>2015<br>USA	Describe types of current activity involvement and barriers to activities as perceived by nursing home residents with dementia.	Qualitative<br>Interviews with care home residents with dementia	NA	Participants primarily depended on activities organized by the facilities and felt they had limited opportunities for activities. Environmental factors, along with a fixed activity schedule, prevented them from engaging in activities.
Voelkl<br>2003<br>USA	Examine the use of the nursing home environment by residents and staff and staff perceptions as to the predictors, barriers and affordances of residents' engagement in activities when in the public environments of the nursing home.	Mixed- Method<br>Observational Data Collection.<br>Three focus groups: one with residents, one with nursing staff, and one with recreation staff.	One nursing home	Observation showed that residents were most frequently engaged in either eating/drinking or in no observable behavior. Staff most frequently were observed to be talking, traveling, or cleaning.<br>Focus group data indicated that resident characteristics and the facility schedule predicted whether or not residents spent time in the nursing home's public environments. Barriers to engagement included management, physical environment, resident characteristics, staff philosophy, and resident perceptions.
Wood<br>2005<br>USA	Examine associations among routine activity situations on a special care unit and how seven residents with moderate to severe Alzheimer’s occupied their time across the day, interacted with other persons and physical environmental elements, and exhibited a range of affective expressions.	Quantitative<br>Activity in Context and Time	One nursing home	Although meals/snacks and some activity groups were positively associated with use of physical objects and engagement in activities, residents were predominantly environmentally disengaged, inactive, or without positive affects during the most prevalent activity situations of background media, downtime, and television.

Woodhall<br>2024<br>UK	Explore how close family members perceive themselves to contribute to the occupational experiences of relatives who are aged 65 or over, have a diagnosis of dementia and live within UK-based care homes.	Qualitative<br>Semi-structured interviews with five family members	NA	Close family members contributed to their relatives' occupational experiences by<br>• Being vigilant and advocating to rectify perceived deficits in care home staff's provision of activity<br>• Providing their relatives with personalised, hands-on support to access more frequent and higher-quality opportunities for occupational participation and engagement<br>• Maintaining their relatives' occupational identity.
Yous<br>2023<br>Canada	Describe environmental, social, and sensory factors influencing meaningful engagement of persons with advanced dementia during Namaste Care implementation in LTC.	Qualitative<br>Focus groups and interviews with staff members	Two care homes	Environmental Attributes<br>• Having a dedicated and quiet space, residents required their own private space away from usual distractions within LTC homes.<br>• Namaste Care should not be delivered in common areas, such as the dining room, or next to noisy environments, such as the nursing station.<br>• A small group setting enhances opportunities for companionship.<br>Social Attributes<br>• Capacity of Namaste Care staff<br>• Families provide important information that can help tailor activities to residents' needs and abilities.<br>Sensory Attributes<br>• Activities selected should provide comfort and distractions for residents.
